# Application of Cognitive Bias Testing in Neuropsychiatric Disorders: A Mini-Review Based on Animal Studies

**DOI:** 10.3389/fnbeh.2022.924319

**Published:** 2022-07-01

**Authors:** Yu-Han Zhang, Ning Wang, Xiao-Xiao Lin, Jin-Yan Wang, Fei Luo

**Affiliations:** ^1^CAS Key Laboratory of Mental Health, Institute of Psychology, Chinese Academy of Sciences, Beijing, China; ^2^Department of Psychology, University of Chinese Academy of Sciences, Beijing, China

**Keywords:** cognitive bias test, animal research, affective state, application, memory bias, interpretation bias

## Abstract

Cognitive biases can arise from cognitive processing under affective states and reflect the impact of emotion on cognition. In animal studies, the existing methods for detecting animal emotional state are still relatively limited, and cognitive bias test has gradually become an important supplement. In recent years, its effectiveness in animal research related to neuropsychiatric disorders has been widely verified. Some studies have found that cognitive bias test is more sensitive than traditional test methods such as forced swimming test and sucrose preference test in detecting emotional state. Therefore, it has great potential to become an important tool to measure the influence of neuropsychiatric disorder-associated emotions on cognitive processing. Moreover, it also can be used in early drug screening to effectively assess the potential effects or side effects of drugs on affective state prior to clinical trials. In this mini-review, we summarize the application of cognitive bias tests in animal models of neuropsychiatric disorders such as depression, anxiety, bipolar disorder, and pain. We also discussed its critical value in the identification of neuropsychiatric disorders and the validation of therapeutic approaches.

## Introduction

Emotions can cause the brain to distort the truth, leading to a discrepancy between what we believe is true and reality. Cognitive bias is the tendency of the brain to process information in favor of certain emotional valence (Lovibond and Lovibond, 1995). Positive emotions lead to positive cognitive biases, while negative emotions cause negative biases, affecting multiple cognitive processes such as attention, memory, and decision-making (Everaert et al., 2012). The phenomenon of cognitive bias is widespread, especially in neuropsychiatric disorders. The concept of “cognitive bias” was first proposed by Beck in the study of patients with depression (Beck, 1967). Based on Beck’s theory, early adverse experiences can trigger negative cognitive schemas leading to negative views of the self, the world, and the future, which in turn lead to biases in cognitive processing (Segal, 1988). According to Bower’s theory of mood congruity (Bower, 1981), during cognitive processing, individuals tend to focus, process, and recall information that is consistent with their emotional state, resulting in cognitive biases.

Cognitive biases can be divided into three types: attentional bias, interpretation bias, and memory bias. Attentional bias indicates that individuals are more likely to allocate attention to stimuli consistent with their current emotional state (Mennen et al., 2019). In animal research, attentional bias can be investigated by analyzing the behavioral response to threatening stimuli (Lee et al., 2016; Luo et al., 2019). Interpretation bias affects decision-making processes. Individuals are more likely to interpret ambiguous cues to be consistent with their current affective state (Everaert, 2021). Interpretation bias in animal research is often measured using the judgment bias test (JBT) (Nguyen et al., 2020), which relies on certain behaviors (like bar-pressing) and these results are then interpreted with respect to certain human constructs, one of them being “attitude” (see more details in Table 1). For example, animals in a more positive affective state tend to interpret ambiguous cues in a more positive way. Memory bias is most often measured through the affective bias test (ABT) and the modified affective bias test (mABT) in animals (Mitte, 2008). The ABT is based on the assumption that emotional state during the memory coding stage affects the perception of reward value (Stuart et al., 2013), while the mABT examines the ability of an animal to form memory bias based on reward value (Stuart et al., 2015).

**TABLE 1:** Some methodological details of representative cognitive bias paradigms.

Paradigm	Stimuli/Cues	Reward	Punishment	Training duration	Testing duration	References
Auditory judgment bias test	Tones	Sweetened condensed milk	Electric shock	3 phases, 21−27 days, 1 session/day, 30 min or 20 trials/session	6 days, 1 session/day, 23 trials/session	Enkel et al., 2010
		One food pellet	Air-puff	3 phases, 15−40 days, 1 session/day	40 min or when 66 trials were completed	Jones et al., 2018
				3 phases, 16−22 days, 1 session/day	40 min or when 60 trials were completed	
		Four reward pellets (high reward); one reward pellet (low reward)	\	4 phases, 23−29 days, 1 session/day, maximum 100 trials or 60 min/session	2 sessions of 100 trials, 2 sessions of 120 trials, 1 session/day	Hales et al., 2020
Spatial judgment bias test	Positions	Overhead light off and 20 mg chocolate flavored pellet paired with one arm	Overhead light on paired with another arm	6 days, 10 min/day	10 min	Novak et al., 2015
		One food pellet (high reward); one quinine-soaked pellet (low reward)	\	2 days, 12 trials/day	3 days, 13 trials/day	Burman et al., 2009
Tactile judgment bias test	Sandpapers	Chocolate (high reward); cheerio (low reward)	\	Minimum 10 days, 4 trials/day	5 days, 4 trials/day	Brydges et al., 2012
Visual judgment bias test	Bars	Sweet condensed milk	Houselight on	9 phases, 89−111 days, maximum 50 trials/day	5 days, 50 trials/day and no more than 30 min/day	Krakenberg et al., 2019
Olfactory judgment bias test	Scents	Dried, sweetened banana chips (high reward), regular rodent chow (low reward)	\	3 phases, 17−19 days, 2−4 trials/day	3 trials, 2 min/trial	Resasco et al., 2021
Affective bias test	Substrates	One sugar tablet	\	5 days	5 days, including 4 days for reward pairing, and 1 day for preference testing (1 session, 30 trials)	Stuart et al., 2013, 2015
Modified affective bias test	Substrates	Two sugar tablets (high reward); one sugar tablet (low reward)	\	*5 days	5 days, including 4 days for reward pairing, and 1 day for preference testing (1 session, 30 trials)	Stuart et al., 2013; Hinchcliffe et al., 2017

*In the current studies, the modified affective bias test is often carried out after the affective bias test, therefore no additional training is required before testing.

Animal experiments are an important complement to human research, especially in the study of neurological and psychological phenomena. Animal research has unique advantages to investigate the underlying mechanisms of these phenomena. For ethical considerations, pharmacological, genetic, and invasive human research is greatly limited, while neurophysiological methods that simulate abnormal states and pharmacological experiments in animals can be conducted to explore specific brain regions, neurons, and even molecules, to better understand the mechanisms behind phenomena, leading to targeted interventions. Harding et al. (2004) were the first to use cognitive bias testing in animals. The presented mini-review briefly summarizes the application of cognitive bias tests in animal research to further explore cognitive bias alterations in neuropsychiatric disorders and the neuropsychological mechanism of cognitive bias, which can ultimately lead to the early identification and treatment of these disorders.

## Application of Animal Cognitive Bias Testing

### Cognitive Bias in Neuropsychiatric Disorders

Many neuropsychiatric disorders are accompanied by emotional alterations which in turn can lead to cognitive biases. One application of cognitive bias testing is to reflect the affective state under different disorders. Currently, cognitive bias tests have been applied in animal models of depression, anxiety, bipolar disorder, and pain (see more details in Table 2). The next section briefly discusses the application of cognitive bias tests in some disorders.

**TABLE 2:** Cognitive bias in animal models of neuropsychiatric disorders.

Models of neuropsychiatric disorders	Animals	Gender	Paradigm	Bias
**Depression**				
Chronic psychosocial stress: daily social defeat for 3 weeks	Sprague Dawley rats	Male	Auditory judgment bias test	Negative; Papciak et al., 2013
Chronic restraint stress: 1-h daily immobilization for 3 weeks	Sprague Dawley rats	Male	Auditory judgment bias test	Negative; Rygula et al., 2013
Chronic unpredictable mild stress (CUMS): Both physical and social stressors were presented randomly across the light/dark cycle	Long-Evans rats	Male	Tactile judgment bias test	Negative; Chaby et al., 2013
Early life adversity: Maternal separation	Sprague Dawley rats	Male	Auditory judgment bias test;	Non-significant; Stuart et al., 2019
			Affective bias test; Modified affective bias test	More prone to corticosterone induced negative bias; A significant deficit in reward-associated positive bias; Stuart et al., 2019
Early life adversity: Early life competition	European starlings	Male and female	Visual judgment bias test	Negative; Bateson et al., 2015
Genetic model: 5-HTT knockout	Wildtype (+/+), heterozygous (+/−), and homozygous (−/−) 5-HTT knockout mice	Female	Spatial judgment bias test	No significant difference between the three groups; Kloke et al., 2014
Genetic model: Learned helpless model	Congenitally helpless (cLH) and congenitally non-helpless (cNLH) rats	Male	Spatial judgment bias test	More negative in cLH rats than that in cNLH rats; Enkel et al., 2010; Richter et al., 2012
			Auditory judgment bias test	
5-HT depletion model: Para-chlorophenylalanine (pCPA) (50 mg/kg) for 6 days	German Landrace piglets	Female	Spatial judgment bias test	Negative; Stracke et al., 2017
Exposure to an isolation stressor of 60 min	Gallus	Male	Visual judgment bias test	Negative; Salmeto et al., 2011; Hymel and Sufka, 2012
**Anxiety**				
Change in light levels: Switch from low to high light levels	Lister-hooded rats	Male	Spatial judgment bias test	Negative; Burman et al., 2009
Light stimuli: Red or white light	BALB/c mice	Male	Olfactory judgment bias test	Negative bias in white light than in red light; Boleij et al., 2012
Anxiogenic drug FG7142 (3.0, 5.0 mg/kg)	Lister-hooded rats	Male	Auditory judgment bias test	Negative; Hales et al., 2016
FG7142 (1.0, 3.0, 5.0 mg/kg)	Lister-hooded rats	Male	Affective bias test	Negative in 3.0, 5.0 mg/kg and non-significant in 1.0 mg/kg; Stuart et al., 2013
FG7142 (5.0 mg/kg)	Lister-hooded rats	Male	Affective bias test	Negative; Stuart et al., 2015
FG7142 (3.0, 6.0 mg/kg)	Sprague Dawley rats	Male	Affective bias test	Negative; Hinchcliffe et al., 2017
Exposure to an isolation stressor of 5 min	Gallus	Male	Visual judgment bias test	Negative; Salmeto et al., 2011; Hymel and Sufka, 2012
1-methyl-chlorophenylpiperazine(m-CPP) (2 mg/kg)	Merino sheep	Female	Attention bias test	Negative; Lee et al., 2016
**Bipolar disorder and Mania**				
D-amphetamine (2 mg/kg) for 2 weeks	Sprague Dawley rats	Male	Auditory judgment bias test	Non-significant; Rygula et al., 2015b
*D-amphetamine (0.1, 0.5, 1 mg/kg)	Sprague Dawley rats	Male	Auditory judgment bias test	Positive in 1 mg/kg and non-significant in 0.1 and 0.5 mg/kg; Rygula et al., 2014
*Amphetamine (0.1, 0.3 mg/kg)	Lister-hooded rats	Male	Auditory judgment bias test	Positive in 0.3 mg/kg and non-significant in 0.1 mg/kg; Hales et al., 2017
**Pain**				
Chemotherapy-induced mucositis: Fluorouracil (5-FU) (150 mg/kg)	Sprague Dawley rats	Male	Tactile judgment bias test	Negative (72 h post 5-FU injection) and non-significant (120 h post 5-FU injection); George et al., 2018
Partial saphenous nerve injury (PSNI)	Lister-hooded rats	Male	Affective bias test;	Negative bias was corrected by gabapentin; 50 mg/kg; Phelps et al., 2021
			Modified affective bias test	A significant deficit in reward-associated positive bias; Phelps et al., 2021
Postoperative pain: Hot-iron disbudding	Holstein calves	Male	Visual judgment bias test	Negative; Neave et al., 2013
Tumors transplantation	Nude mice	Male	Olfactory judgment bias test	Negative; Resasco et al., 2021
		Female		Non-significant; Resasco et al., 2021

*Acute administration of amphetamines may simply induce hyperactivity rather than strictly mania.

#### Depression

Depression is a mood disorder accompanied by low self-esteem, impaired cognitive function, and decreased pleasure (Monroe and Anderson, 2015). In human studies of cognitive bias, it was found that depressed subjects are more inclined to focus on negative stimuli (Armstrong and Olatunji, 2012), choose more negative words as self-descriptive (Dainer-Best et al., 2018), and recall more negative items and less positive items on memory tests (Bianchi et al., 2020). Harding et al. were the first to apply the judgment bias paradigm to investigate the cognitive bias of rats (Harding et al., 2004), demonstrating that the JBT can be used to detect negative emotions in animals.

Animal models of depression include chronic stress, learned helplessness, deficits in the serotonin system, and adverse experiences in early life (Czéh et al., 2016). Rats exposed to chronic physical stress or chronic psychosocial stress negatively interpret ambiguous cues, approach rewards more slowly, and experience a series of long-term cognitive and behavioral changes (Salmeto et al., 2011; Hymel and Sufka, 2012; Chaby et al., 2013; Papciak et al., 2013). Compared with congenitally non-helpless rats, congenitally helpless rats showed decreased positive responses and increased negative responses to ambiguous cues (Enkel et al., 2010; Richter et al., 2012). A study found that inhibiting serotonin synthesis through para-chlorophenylalanine (pCPA) dosing in pigs leads to a shift to more pessimistic judgments of ambiguous stimuli (Stracke et al., 2017). Results from early adverse experience models have shown lowered expectation of reward in response to ambiguous information (Bateson et al., 2015). Of particular interest, Stuart et al. (2019) found that rats experiencing maternal separation were more prone to corticosterone-induced negative bias and showed a deficit in reward-associated positive bias in mABT, whereas no significant difference was found in the sucrose preference test. This finding indicates that cognitive bias testing is a sensitive and important tool in depression-like state assessment.

Forced swimming test, sucrose preference test, and open-field test are widely used in animal studies to detect depression-like behaviors such as behavioral despair, anhedonia, and exploratory behaviors (Hu et al., 2017). These tests do not require training, while cognitive biased tasks require long-term and complex conditional training, as shown in Table 1. Although the cognitive bias test needs more experimental efforts, the affective bias measured by it could not be replaced by other tests (Robinson, 2018). Therefore, cognitive bias test can be used as a good supplement to the commonly used depression-like behavior test and plays a unique role in mechanism research (Stuart et al., 2015) and drug screening (Stuart et al., 2017).

#### Anxiety

Negative cognitive biases induced by anxiety can help an organism attend to threatening stimuli quickly, leading to an avoidance of potential danger. In a human study, it was found that anxious subjects exhibit an exaggerated attentional bias toward threats and overestimate detrimental consequences of events (Aue and Okon-Singer, 2015). In a JBT study of chicks under anxiety-like state, more pessimistic-like approach behaviors were exhibited to ambiguous aversive cues (Salmeto et al., 2011; Hymel and Sufka, 2012). Using pharmacological methods, one study found that sheep injected with the anxiety-stimulating drug 1-methyl-chlorophenylpiperazine (m-CPP) show increased attention toward threats accompanied by increased vigilance (Lee et al., 2016), leading to negative attentional bias. Other studies found that acute injection of anxiogenic drug FG7142 in rats led to negative cognitive bias in both judgment bias tests (Hales et al., 2016) and affective bias tests (Stuart et al., 2013, 2015; Hinchcliffe et al., 2017).

Studies have shown that high-intensity light and white light are aversive to rodents, while dim light and red light are more neutral (Burman et al., 2009; Boleij et al., 2012) and therefore, alterations in lighting can be used to manipulate anxiety level in rodents. There is strong evidence that rats trained in dim lighting conditions but tested in bright lighting conditions have longer approach latencies when exposed to ambiguous cues (Burman et al., 2009; Boleij et al., 2012), indicating that acute increase in anxiety leads to negative judgment bias.

#### Bipolar Disorder and Mania

Depression and mania are the two core components of bipolar disorder. The cognitive and emotional correlates of depression have been extensively studied, but related research on mania is relatively lacking. Chronic administration of the psychostimulant d-amphetamine has been used to cause manic-like symptoms in animals (Valvassori et al., 2019). Some studies have shown that acute d-amphetamine administration can induce an optimistic bias in rats (Rygula et al., 2014; Hales et al., 2017), while another study found that two consecutive weeks of amphetamine treatment does not cause significant positive bias (Rygula et al., 2015b). However, it is not clear whether acute administration of amphetamines induces a manic-like state or simply a state of hyperactivity (Minassian et al., 2016).

In clinics, the mood stabilizers lithium and valproate are the most commonly used drugs to treat bipolar disorder (Geddes and Miklowitz, 2013). They can help patients find a balance between depression and mania (McIntyre et al., 2020). An animal study found that acute administration of lithium induced optimistic bias in rats that were generally pessimistic, while no significant bias was observed after injection of valproic acid in rats that were more neutral at baseline, which suggests that the effect direction of lithium may be affected by the valence of cognitive bias (Rygula et al., 2015a). Although such studies are rare, it still suggests that cognitive bias tests have the potential to be applied to the animal study of pharmacological mechanisms associated with bipolar disorder.

#### Pain

Pain includes not only physiological components but emotional and cognitive components as well (Price, 2000). Pain in humans can lead to decreased quality of life, anxiety, and depression (Kendig et al., 2000), while pain in animals can lead to reduced water and food intake and abnormal grooming, nesting, and burrowing behaviors (Jirkof, 2017). Previous studies have frequently used conditioned place avoidance (CPA) to examine emotion and avoidance associated with pain (Tappe-Theodor et al., 2019). However, the emotional and cognitive components of pain may be more complex. Cognitive bias tests, such as the JBT, focus on animals’ interpretation of ambiguous information, while the ABT includes reward value. Therefore, cognitive bias tests will help to explore the emotion-motivation and cognition-evaluation dimensions of pain from diverse perspectives.

Dairy calves experiencing postoperative pain associated with hot-iron disbudding to prevent horn growth exhibited a negative interpretation of ambiguous cues (Neave et al., 2013). A study on rats with chronic inflammatory pain as a result of 5-fluorouracil (5-FU) injection to simulate chemotherapy-induced intestinal mucositis, found that 72 h after injection, optimistic decision-making was significantly reduced (George et al., 2018), while 120 h after injection, optimistic decision-making increased as the damaged intestine gradually recovered (George et al., 2018). Chronic neuropathic pain caused by saphenous nerve injury leads to a negative bias which can be corrected by gabapentin as tested by the ABT, and a reward deficit in developing value-based memory bias in the mABT (Phelps et al., 2021), suggesting that rats with chronic neuropathic pain experience negative emotions and deficits in sensitivity to reward value. In addition, a study using the JBT to examine cancer pain and discomfort in mice with tumors found that tumor-bearing male mice were more pessimistic than healthy controls (Resasco et al., 2021). In sum, these studies indicate that cognitive bias tests can effectively measure the negative emotional state caused by pain in animals from acute pain to chronic pain and that analgesics can partially correct this state, therefore can be used in the validation of therapeutic approaches.

### Cognitive Bias Tests in Assessing the Effect of Drugs on Affective State

Cognitive bias tests have shown good validity in the assessment of drug-induced affective changes (Robinson, 2018), providing a new approach for preclinical drug screening. Studies using the ABT found that acute administration of the antidepressants such as fluoxetine, reboxetine, venlafaxine, and mirtazapine induced positive biases in animals (Hales et al., 2017). However, one problem with the ABT and other preclinical testing methods, such as forced swimming, is the inability to distinguish between acute and delayed onset of antidepressant action. For example, fluoxetine was found to act quickly in preclinical trials using forced swimming, but with delayed clinical onset (Cryan and Holmes, 2005). The JBT can help to resolve this issue. Acute administration of the conventional antidepressants fluoxetine, reboxetine, or venlafaxine did not cause an interpretation bias in animals compared to the clinical fast-acting antidepressant ketamine, and only long-term use of fluoxetine resulted in a positive bias (Hales et al., 2017). These data indicate that the JBT better reflects the time course of antidepressant effects and effectively screens out fast-acting drugs at the preclinical stage.

Negative emotional side effects caused by drugs can greatly reduce a patient’s quality of life, affect medication compliance, and even cause the original therapeutic regimen to be broken down (George et al., 2018). Therefore, it is critical to assess potential emotional side effects of medication during preclinical studies. Cognitive bias tests have been used to study the emotional side effects of medications. One study used ABT to test some drugs that can increase the risk of depression in clinical patients and found that lipopolysaccharides (LPS), interferons-alpha (IFN-α), and tetrabenazine (a drug for the treatment of chorea in Huntington’s disease) (Frank, 2010) can induce negative deviation in rats, but varenicline (a smoking cessation drug) (Tonstad et al., 2020), carbamazepine (an anticonvulsant) (Israel and Beaudry, 1988), or montelukast (an anti-asthma drug) (Markham and Faulds, 1998) did not induce significant bias (Stuart et al., 2017). At present, the JBT has not been widely used in the preclinical screening of emotional side effects of drugs due to its long training time and complexity. It is necessary to further develop a more sensitive, fast, and simple animal experimental paradigm for cognitive bias in future research.

## Discussion

An important interpretation for the behavioral results of cognitive bias test is to reflect the emotional state of animals and its effectiveness has been widely verified (Nguyen et al., 2020), indicating potential application in animal studies associated with neuropsychiatric disorders. Compared to the forced swimming test, the JBT is more sensitive to the clinical onset time of antidepressants, while the ABT is more sensitive in the assessment of reward deficits than the sucrose preference test. Therefore, cognitive bias tests may be used for the early identification of neuropsychiatric disorders and validation of their therapies.

It should be mentioned that in addition to the change of emotional state, motivation factors can also affect cognitive bias. For example, Enkel et al. (2010) noticed that in different depression-like states, a pessimistic judgment bias toward ambiguous cues could result from a decrease in positive response rate coupled with either (1) an increase in negative response rate or (2) an increase in omission rate. The former may reflect increased motivation to avoid potential punishment, whereas the latter may reflect decreased motivation to approach potential reward. This indicates that even in similar affective states, different motivational mechanisms may underlie the formation of bias. Due to the length of the min-review, we cannot discuss more, but we refer interested readers to the review by Lewis et al. (2019) and a recent paper by Neville et al. (2020), both of which provide an in-depth discussion on this topic.

The psychological mechanisms underlying the emergence and transition of cognitive bias remain unclear. One theory explains the emergence of cognitive bias from the perspective of biological evolution and adaptation (Durisko et al., 2015). In everyday life, most information is ambiguous with few explicit cues. Therefore, individuals must use prior experiences to interpret the meaning of current situation ambiguous cues (Norbury et al., 2018). This cognitive process is vital to animal survival and is an adaptive behavior that can be influenced by cognitive bias, which can be advantageous in limiting cognitive resources for faster and more efficient decision-making (Enkel et al., 2010). However, in some disorders, cognitive bias may remain constant, leading to non-adaptive behaviors. For example, negative cognitive biases associated with depression are developed by exposure to persistent stress and other adverse factors. These negative cognitive biases lead to risk-avoidance and loss-reducing behavioral strategies (Durisko et al., 2015) which can be advantageous in an unsafe environment. However, in a safe environment, these behaviors can be non-adaptive. A depressed individual may not have the capacity to alter negative biases in different situations. The ability to alter biases to appropriately address the presented situation needs further research.

Precision medicine is a hot spot in clinical research in recent years (Manchia et al., 2020). The detection of individual emotional characteristics will help to formulate an individualized treatment plan for emotional diseases. Prior studies have shown that the effects of acute manipulation of the dopamine and serotonin systems on cognitive bias may depend on cognitive bias baseline. After acute administration of haloperidol, a dopamine D2 receptor antagonist, or escitalopram, a 5-HT reuptake inhibitor, “optimistic” rats became more pessimistic, while “pessimistic” rats became more optimistic (Golebiowska and Rygula, 2017a). Therefore, cognitive bias tests may serve to formulate therapeutic regimens based on individual patient characteristics and, as such, should be included in future neuropsychiatric drug research.

Finally, the neural mechanisms of cognitive biases are understudied. The prefrontal area plays an important role in decision-making under ambiguity and risk (Rouault et al., 2019). A study in rats found that lesions to the orbitofrontal cortex (OFC) but not to the medial PFC (mPFC) decreased the proportion of positive lever presses and increased the proportion of negative lever presses in response to ambiguous tones, indicating increased pessimism (Golebiowska and Rygula, 2017b). The basolateral amygdala is closely associated with prefrontal regions and is also involved in the assessment of ambiguity and uncertainty (Davis and Whalen, 2001). One study found that unpredictability increased c-Fos expression in the lateral amygdala of mice (Herry et al., 2007). Likewise, the lateral septum is an important area for the integration of cognitive and affective information that compares known information with unknown and inferred ambiguous cues (Wirtshafter and Wilson, 2021). A study has shown a decrease in c-Fos expression in the lateral septum in response to ambiguous cues (Boleij et al., 2012). Further research using surgery, electrophysiology, optogenetics, *in vivo* calcium imaging, and other techniques to study the neural correlates of cognitive bias is necessary to identify key brain regions and molecular targets of potential therapeutics.

## Author Contributions

NW, J-YW, and FL contributed to the conception of the review. Y-HZ wrote the original draft of the manuscript. NW and X-XL wrote sections of the manuscript. All authors contributed to manuscript revision, read, and approved the submitted version.

## Conflict of Interest

The authors declare that the research was conducted in the absence of any commercial or financial relationships that could be construed as a potential conflict of interest.

## Publisher’s Note

All claims expressed in this article are solely those of the authors and do not necessarily represent those of their affiliated organizations, or those of the publisher, the editors and the reviewers. Any product that may be evaluated in this article, or claim that may be made by its manufacturer, is not guaranteed or endorsed by the publisher.
